# An efficient and stable photoelectrochemical system with 9% solar-to-hydrogen conversion efficiency via InGaP/GaAs double junction

**DOI:** 10.1038/s41467-019-12977-x

**Published:** 2019-11-21

**Authors:** Purushothaman Varadhan, Hui-Chun Fu, Yu-Cheng Kao, Ray-Hua Horng, Jr-Hau He

**Affiliations:** 1 Computer, Electrical, and Mathematical Sciences and Engineering, King Abdullah University of Science and Technology, (KAUST), Thuwal, 23955-6900 Saudi Arabia; 2 KAUST Solar Center, KAUST, Thuwal, 23955-6900 Saudi Arabia; 3 Graduate Institute of Precision Engineering, National Chung Hsing University, Taichung, 402 Taiwan, ROC; 4 Institute of Electronics, National Chiao Tung University, Hsinchu, 300 Taiwan, ROC; 5 Center for Emergent Functional Matter Science, National Chiao Tung University, Hsinchu, 300 Taiwan, ROC; 6 Department of Materials Science and Engineering, City University of Hong Kong, Kowloon, Hong Kong

**Keywords:** Devices for energy harvesting, Artificial photosynthesis, Solar fuels

## Abstract

Despite III-V semiconductors demonstrating extraordinary solar-to-hydrogen (STH) conversion efficiencies, high cost and poor stability greatly impede their practical implementation in photoelectrochemical (PEC) water splitting applications. Here, we present a simple and efficient strategy for III-V-based photoelectrodes that functionally and spatially decouples the light harvesting component of the device from the electrolysis part that eliminates parasitic light absorption, reduces the cost, and enhances the stability without any compromise in efficiency. The monolithically integrated PEC cell was fabricated by an epitaxial lift-off and transfer of inversely grown InGaP/GaAs to a robust Ni-substrate and the resultant photoanode exhibits an STH efficiency of ~9% with stability ~150 h. Moreover, with the ability to access both sides of the device, we constructed a fully-integrated, unassisted-wireless “artificial leaf” system with an STH efficiency of ~6%. The excellent efficiency and stability achieved herein are attributed to the light harvesting/catalysis decoupling scheme, which concurrently improves the optical, electrical, and electrocatalytic characteristics.

## Abstract (web summary)

Photoelectrochemical water-splitting devices with III-V semiconductors are efficient for solar-to-hydrogen conversion, but high costs and poor stability limit applications. Here, authors decouple light harvesting from electrolysis to enhance stability without compromising the efficiency.

## Introduction

Solar energy is intermittent in nature, creating an inherent mismatch between photovoltaic (PV) energy production and consumption, and limiting the degree to which we can depend on it for the electrical grid^1–4^. In this regard, solar-driven water-splitting is a viable alternative that has the potential to realize affordable solar fuels by converting solar energy into a storable power source, such as hydrogen^2–8^. Among various material systems considered for photoelectrochemical (PEC) solar fuel generation^9–12^, III-V semiconductors have received significant attention because of their appropriate bandgap and electronic and transport properties, which are suitable for PEC water-splitting applications^13–16^. In particular, tandem systems composed of InGaP/GaAs double junction photoelectrodes have the potential to drive unassisted PEC water-splitting, with solar-to-hydrogen conversion (STH) efficiencies of 10–19%^14–20^. Despite their excellent photophysical properties and record high efficiencies, the high cost and poor PEC stability prevent their real-world applications.

In particular, III-V semiconductors spontaneously photocorrode in electrochemical conditions necessary for water splitting, leading to rapid deterioration and catastrophic device failure^19^. Recently, Young et al. have demonstrated an STH efficiency of up to 16% using InGaP/InGaAs tandem double junction photoabsorbers, though with a stability of <1 h, illustrating the necessity of engineering novel surface protection strategies^20^. Extensive research efforts have been devoted over the past decade on surface protection and passivation materials for III-V and other photoelectrodes by the deposition of transition metals, metal oxides, or metal silicides, etc.^17,19,21–26^. These efforts have led to partial success in providing improved stabilities and lifetimes, yet most of these devices fail within a few minutes, and with few lasting up to several hours^19^.

At present, almost all PEC systems (e.g., Si and III-Vs) possess the classical monofacial device configuration, in which all the major device components, including the light absorber, surface passivation or protection layer, and electrocatalysts, are integrated on one side of the device, whereas the other side can be used as a carrier collector^15,22,27,28^. By nature, this device configuration requires stringent prerequisites, such as optimized band alignment, thin and transparent surface protection layers, and electrocatalysts. The integration of the surface protection layers on top of the light-absorbing layer creates a trade-off between the surface protection, layer thickness, carrier selective conductivity, optical transparency, and the band alignment, which further limit the ability to optimize the surface protection characteristics^13,28^. Similarly, most electrocatalysts are non-transparent, and in particular earth-abundant electrocatalysts require higher loading capacities to reduce the overpotentials^20,29^, which introduces more parasitic light blocking. In simple terms, most photoelectrodes are monofacially functional, which means one side of the device is overused while the other side is underutilized^21^. Consequently, there are insufficient options for further optimization of the cost, performance, and stability of current single sided PEC devices.

Such issues can be addressed by employing an innovative scheme of decoupling the optical absorption and electrocatalytic interfaces to synergistically enhance the optical, electrical, surface protection, and electrocatalysis of the overall PEC system. In this design, the top surface of the photoelectrode is engineered to maximize the light harvesting capacity, whereas the bottom surface is dedicated to carrying out the required electrochemical reactions. Recently, Vijselaar et al. have explored the partial decoupling of light absorption and electrocatalysis through the spatioselective deposition of electrocatalysts on high-aspect-ratio Si microstructures^28^, but such designs are cumbersome and challenging to achieve in scalable and efficient PV-PEC devices.

Another major limitation for the large-scale production of III-V based PEC systems is their vast manufacturing cost, primarily due to the requirement of bulk homo GaAs or Ge substrates (76% of the cost) that are used to achieve the desired lattice-matched epitaxial light-absorbing junctions^30^. The reuse of single crystalline substrates by the epitaxial lift-off (ELO) method to release and transfer the grown light-absorbing III-V layers to a cheap and earth-abundant substrate would be an attractive option for reducing the cost. Rather than simply focusing on the demonstration of record efficiencies, it is necessary to develop such large-scale ELO and transfer processes to reuse the GaAs substrate in conjunction with a surface protection scheme under standard PEC conditions in order to achieve further progress in practical III-V-based PEC water splitting. Furthermore, ELO and transfer onto flexible substrates would provide an opportunity to realize flexible and bendable PEC devices rather than the rigid devices demonstrated thus far. Additionally, achieving a standalone unassisted-wireless device would be attractive, as the absence of external connections would significantly simplify the device design and cost^7,31^. In the case of unassisted-wireless water-splitting, the resultant hydrogen and oxygen evolve at the opposite sides of the device, which makes the product separation easier. However, there are still multiple challenges, such as achieving an electrically conductive front and back side with optimum catalyst integration, and the efficient transport of protons from the anode to the cathode side. All of these issues lead to significant Ohmic losses and diminished overall STH efficiency.

As a solution, we present a stratagem for III-V PEC cells that substantially lowers the cost and increases the stability without compromising the efficiency. Using metalorganic chemical vapor deposition (MOCVD), we obtained a wafer-scale inverted grown InGaP/GaAs double junction, followed by the large-scale deployable ELO and transfer technique, allowing us to recycle the expensive single crystalline GaAs substrate in order to realize a cost-effective III-V-based PEC device. The concurrent improvement in the efficiency and stability of the resultant tandem photoelectrode (InGaP/GaAs/catalyst carrier substrate) is enabled by spatially and functionally decoupling the light absorption and surface protection/catalytic activity, leading to an STH conversion efficiency of ~9% under alkaline electrolyte (0.5 M KOH (aq)). More importantly, by introducing this surface protection strategy, we observe a PEC stability of over 150 h, the highest for any III-V system thus far. Finally, with the ability to access both sides of the device (i.e., the bifacial configuration), we successfully demonstrate the first fully integrated, unassisted wireless III-V-based PEC device, with an STH efficiency of ~6%.

## Results

### Decoupling the optical and electrocatalytic reactive interface

**Fig. 1:**
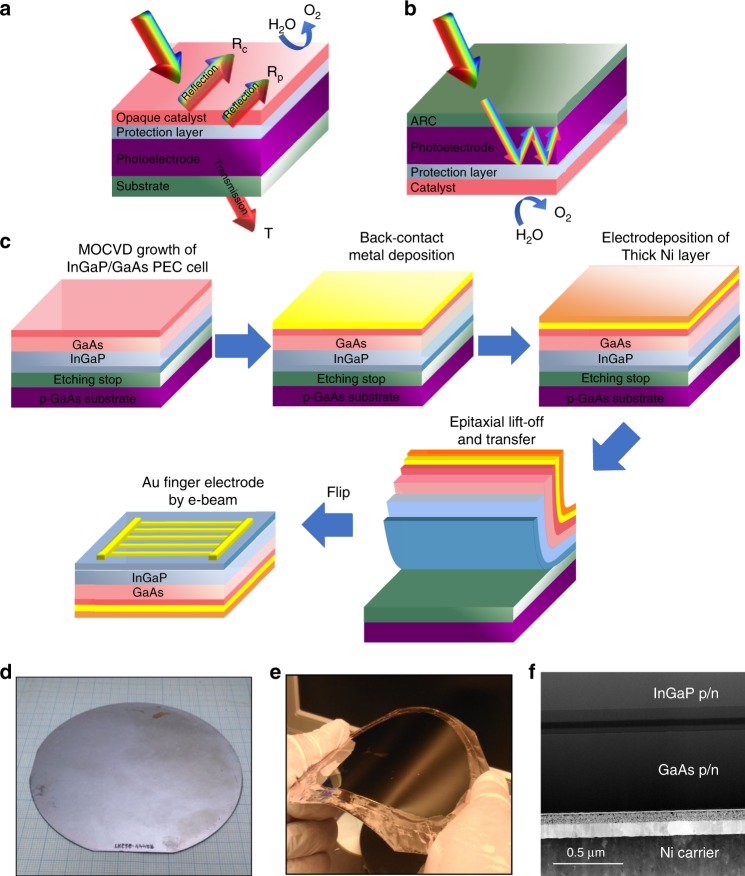
Decoupling optical absorption and electrocatalysis. **a** The typical monofacial PEC device design, in which all functionalities, such as the surface protection layer and electrocatalyst, are integrated on top of the light harvesting side, whereas the bottom side is only used for the electrical connection. The opaque catalytic reflection (Rc) and protection layer reflection (Rp) significantly block light absorption in current monofacial PEC devices. **b** Our novel, light-decoupled device, in which the light harvesting component is decoupled from the surface protection and electrocatalysis functions. **c** Schematic illustration of the steps involved in the ELO and transfer processes to fabricate the integrated InGaP/GaAs tandem photoanode on a flexible substrate for PEC water splitting. **d**, **e** A wafer-scale inversely grown InGaP/GaAs on single crystalline GaAs substrate before (**d**) and after (**e**) ELO and transfer onto a flexible Ni carrier wafer. **f** Cross-sectional TEM image of the transferred tandem photoanode on the Ni substrate

Figure 1a shows the schematic illustration of a typical monofacial PEC device, in which the surface protection layer and electrocatalyst are integrated on the light harvesting side, thus limiting the efficiency and stability. In contrast, Fig. 1b shows our proposed functionality-decoupled device, in which the light harvesting component is decoupled from the surface protection and electrocatalysis functions. Such an inverted structure (Fig. 1b) has distinct advantages over traditional, upright growth (Fig. 1a), which are discussed below. First, the typically opaque protection/electrocatalyst layers exhibit undesirable effects of parasitic light absorption (catalytic reflection (Rc) and protective layer reflection (Rp), as shown in Fig. 1a), leading to decreased photocurrent during PEC water splitting. Next, it is well known that the conventional tandem device loses a portion of the near-band-edge photons (denoted as “T” in Fig. 1a) passing through the GaAs substrate, making the bottom GaAs a current-limiting junction. With our decoupling scheme (Fig. 1b), the light harvesting side can be optimized independently without considering the shadowing effect while the catalyst side can be optimized in terms of catalysis performance and stability. Furthermore, the back reflective catalyst layer can reflect any unabsorbed photons with long wavelengths back, enhancing the long-wavelength photon absorption of the PEC cell.

Though a decoupling scheme is a novel approach for synergistic enhancement of the efficiency, stability, and cost, achieving such decoupling is not straightforward in III-V tandem structures due to the significant optical absorption and lattice mismatch between the sub-cells. To achieve such decoupling, we employed the ELO and transfer method to provide access to the bottom of the PEC device and also recycle the GaAs substrate to significantly reduce the cost (over 50%)^30^. Different from conventional lattice-matched, upright (InGaP/GaAs) tandem PEC cells^27,32^, here we grow PEC cells in an inverted growth architecture (by growing lower bandgap GaAs on top of the higher bandgap InGaP) to realize the upright structure after the ELO and transfer process^33^. In this way, we can achieve high-efficiency tandem double junctions of InGaP/GaAs using conventional epitaxial growth (i.e., MOCVD). A detailed description of the MOCVD growth and ELO and transfer procedure is provided in the Supplementary Information^34^. Figure 1c shows the schematic of the ELO and transfer procedures used to achieve the wafer-scale photoelectrode with the desired substrate. Supplementary Fig. 1 provides a more detailed transfer description of all the layers that make up the device^34^.

Figure 1d, e displays photographs of the wafer-scale InGaP/GaAs tandem junction photoelectrode before and after the ELO and transfer method, respectively, demonstrating the potential for mass production. Figure 1f shows a cross-sectional transmission electron microscopy (TEM) image of the transferred tandem junction on the catalyst layer. It should be emphasized that the wafer-scale ELO method adopted herein has several advantages, including multiple options for the surface protection materials (metals, metal oxides, and others), an earth-abundant electrocatalyst (generally opaque in nature) with loading quantity limited only by cost, maximized photon absorption, and full wafer recycling after lift-off for considerable cost-cutting. Note that micron-scale single-junction GaAs photoelectrodes (250 µm^2^) fabricated via this previously reported ELO and transfer technique^35^ are complex in nature with multiple issues, such as limited options for scalability in combination with poor stability (<8 h), constraining contemporary efforts at a very early proof-of-concept stage.

### Unassisted-wired solar water-splitting

**Fig. 2:**
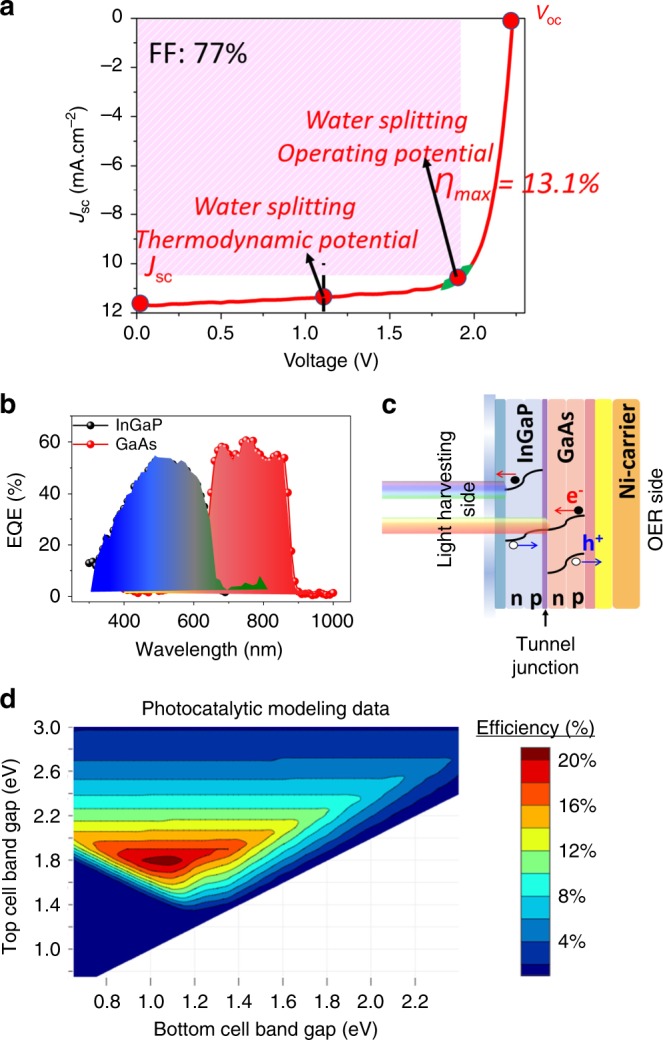
Photovoltaic characteristics and theoretical modeling of the PEC cell. **a** PV characteristics of the inverted InGaP/GaAs double junction, consisting of the top cell InGaP structure (*E*_g_ = 1.8–1.85 eV) and bottom cell GaAs structure (*E*_g_ = 1.4 eV) under one sun AM 1.5 G illumination. **b** EQE measurements and **c** band-diagram of the InGaP/GaAs tandem photoelectrode. **d** Solar fuel efficiency modeling using the bandgap characteristics of the InGaP/GaAs tandem photoelectrodes with suitable electrocatalyst overpotentials

Before characterizing the PEC performance, we measured the PV characteristics of the ELO-transferred InGaP/GaAs double junction, consisting of the top (InGaP, *E*_g_ = 1.8–1.85 eV) and bottom cells (GaAs, *E*_g_ = 1.4 eV) under one sun illumination (Fig. 2a). Under AM 1.5 G simulated sunlight, the short-circuit current density (*J*_SC_), open-circuit voltage (*V*_OC_), and fill factor (FF) were 11.7 mA cm^−2^, 2.25 V, and 0.77, respectively. It should be noted that in tandem cells, the photovoltages of the sub-cells are added together, and therefore the *V*_OC_ achieved is higher than the potential required to drive unassisted water splitting (~1.7–1.8 V).

Figure 2b, c shows the measured external quantum efficiency (EQE) and the corresponding band-diagram of the InGaP/GaAs double junction PEC cell. The EQE of <65% occurs in the wavelength range of 400–900 nm. The cutoff seen below ~375 nm can be explained by the inefficient extraction of carriers generated near the window and emitter layers. By integrating the EQE, we determined the *Jsc* obtained from the top InGaP cell and bottom GaAs cell were 10.6 and 14.7 mA cm^−2^, respectively. The total current generated is limited by the low current from the top InGaP sub-junction (10.6 mA cm^−2^). The total *Jsc* measured under AM 1.5 G illumination was 12.5 mA cm^−2^, and the mismatch in the *Jsc* from the I-V measurement and EQE can be attributed to the imperfect solar spectrum of the solar simulator in the I-V measurement. We also employed solar fuel efficiency modeling to analyze the maximum theoretically achievable PEC efficiency of the photoelectrode (Fig. 2d)^36^. With an appropriate overpotential for the hydrogen evolution reaction (HER) and oxygen evolution reaction (OER) electrocatalysts (100 mV for the HER electrocatalyst (Pt counter electrode) and 300 mV for the OER electrocatalyst (NiO_*x*_)),^37^ a maximum theoretical STH efficiency of 16% can be achieved with the proposed tandem PEC cell.

**Fig. 3:**
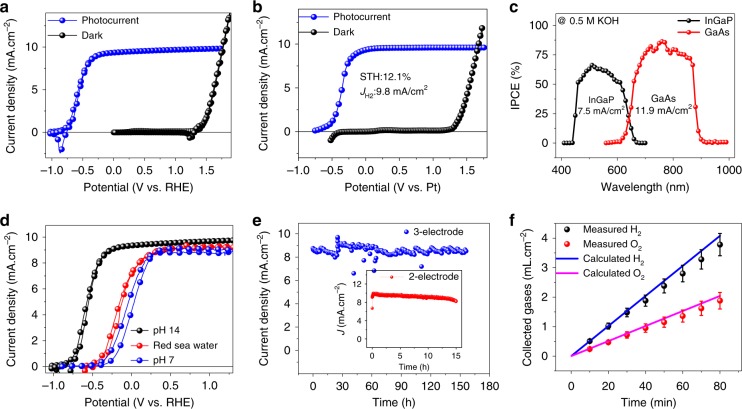
Photoelectrochemical water-splitting characteristics. **a** CV curves of the InGaP/GaAs tandem photoanode measured under a three-electrode system in 0.5 M KOH (aq) electrolyte under one sun illumination and dark electrolysis of the Ni/p^+^Si. **b** LSV curves of the photoanode in 0.5 M KOH (aq) under one sun illumination, in a two-electrode setup. **c** Spectral response of the tandem photoelectrode, for which the integrated light is limiting the current densities under AM 1.5 G illumination. **d** CV curves of the InGaP/GaAs tandem photoanode measured under 0.5 M KOH (pH 14), 1 M Na_2_SO_4_ (pH 7), and as-obtained Red Sea water (pH 8.2). **e** Chronoamperometry of a tandem photoanode in a 3-electrode configuration measured at 0 V vs. RHE under one sun illumination in 0.5 M KOH (aq), demonstrating the stability over 150 h. Inset shows the 2-electrode stability measured by short-circuiting the working and counter electrodes without any applied bias. **f** The measured hydrogen and oxygen as a function of time in a two-cell electrode setup

We characterized the PEC performance of the InGaP/GaAs tandem photoanode using cyclic voltammetry (CV) with a three-electrode setup in a gas-tight PEC quartz cell without any uncompensated resistance (iR) correction (Fig. 3a). The three-electrode measurement setup consisted of Pt as a counter electrode, a saturated calomel electrode (SCE) as a reference electrode, and the tandem PEC cell as the working electrode, using 0.5 M KOH (aq) (pH~13.8) as the electrolyte under one sun AM 1.5 G illumination. During the PEC water-splitting measurements, the light harvesting side was illuminated through a thin quartz window, while the catalyst layer was in contact with the electrolyte where the OER proceeds. It should be noted that the electrochemical OER performance closely matched the PV performance of the device, with a $J_{\mathrm{H}_2}$, $|V_{\mathrm{OS}} - E^o|$ (*V*_OS_ is the onset potential measured at a current density of 1 mA cm^−2^, *E*^0^ is the equilibrium water oxidation potential, and $J_{{\mathrm{H}}_2}$ is the saturation current density at *E*^0^ for OER), and FF of 10.1 mA cm^−2^, 2.0 V vs. RHE, and 0.77, respectively, with a maximum PEC efficiency of 12.4% (Fig. 3a). The excellent $J_{\mathrm{H}_2}$ and $|V_{\mathrm{OS}} - E^o|$ achieved here can be attributed to the decoupling of the light harvesting and electrocatalysis components of the device. More importantly, the resulting $|V_{\mathrm{OS}} - E^o|$ of 2.0 V vs. RHE for the InGaP/GaAs tandem photoanode is optimal to drive unassisted water-splitting under the given experimental conditions (the optimum range is calculated to be 1.9–2.2 V by adding the overpotentials of both the Pt cathode, NiO_*x*_ anode, and other circuit losses)^15,38^. The $|V_{\mathrm{OS}} - E^o|$ of 2.0 V vs. RHE for the PEC cell is ~0.25 V less than the *V*_OC_ measured in air, which is mainly due to electrolytic and interfacial charge carrier recombination losses during OER.

Unassisted wired water-splitting was performed in 0.5 M KOH (aq) by connecting the InGaP/GaAs tandem photoanode to a ∼1.5 cm^2^ Pt deposited Ni foam (as a counter electrode) in a two-electrode setup to form a full PEC cell (Supplementary Fig. 2). Linear sweep voltammetry (LSV) with the two-electrode setup showed a $J_{\mathrm{H}_2}$ of 9.8 mA cm^−2^, with instantaneous bubble formation on both the electrodes, demonstrating the unassisted water-splitting (Fig. 3b). Such two-electrode measurements can be used to calculate the STH efficiency, which is defined as:

1 $$\mathrm{STH}\;\left( {\mathrm{\% }} \right) = \frac{{J_{\mathrm{H}_2} \times (1.23{\mathrm{V}})}}{{P_{\mathrm{in}}}}$$

where *P*_in_ is the total incident light intensity (1000 W/m^2^) under the assumption of 100% Faradaic efficiency. A maximum STH efficiency of ~12% was achieved, which is on par or higher than the efficiency of most III-V-based reports and well above the requirement of 10% STH efficiency postulated by the U.S. Department of Energy for commercial-scale PEC technology^39^. In addition, we measured the $J_{\mathrm{H}_2}$ of the tandem PEC cell vs. the dark cathode (Pt) in a two-electrode system to confirm the claimed efficiency.

To further elucidate the role of the tandem photoelectrode on the PEC performance, we measured the incident-photon-to-current efficiency (IPCE) spectra by short circuiting the working electrode vs. the counter electrode (Pt). The multijunction tandem PEC cells are modeled by sub-cells connected in series, in which every sub-cell has its short-circuit current source. The results reveal a maximum IPCE of 68.5% for the top InGaP cell and 79.9% for the bottom GaAs cell (Fig. 3c). Both values are higher than their corresponding EQE values of about ~10% (Fig. 2b) due to which could be due to the fact that lower reflectance at the electrolyte/semiconductor interface as compared to the semiconductor/air, and the air/glass reflectance. By short circuiting the stack in the tandem PEC cell, the terminal current of every sub-cell will be equal to that of the others. Accordingly, the smallest current will limit the current flow in the stack. As shown in the IPCE spectra (Fig. 3c), the GaAs sub-cell (12.9 mA cm^−2^) (which is typically the current limiting sub-cell in conventional GaAs double junction designs) outperforms the InGaP sub-cell (9.8 mA cm^−2^), demonstrating the effectiveness of our optical and catalytic decoupling scheme in inverted InGaP/GaAs tandem PEC cells. However, the maximum current density obtainable from the InGaP/GaAs photoanode is 7.5 mA cm^−2^, which turns out to be the maximum STH of ~9%. It should be noted that the STH calculated directly from the CV and CA results can have various errors^40^, and hence the STH obtained by integrating the IPCE current density over the reference solar-spectrum is more reliable. Hence the STH efficiency of 9% is more reliable for the PEC cell demonstrated herein.

In order to further evaluate the performance of the InGaP/GaAs tandem photoanode under different electrolytic conditions, we carried out PEC water oxidation under neutral (pH 7, 1 M Na_2_SO_4_), alkaline (pH 14, 0.5 M KOH), and as-obtained Red Sea water (pH 8.2) electrolyte conditions (Fig. 3d). The photoanode exhibits an excellent $J_{\mathrm{H}_2}$ of 10.1 mA cm^−2^ and 8.4 mA cm^−2^ under alkaline and neutral electrolytes, respectively. Even with Red Sea water (to show natural water-splitting), the device exhibits a $J_{\mathrm{H}_2}$ of 8.3 mA cm^−2^_,_ indicating an excellent solar driven water-splitting performance of the integrated PEC cell over a wide range of pH conditions, thereby demonstrating it as a potential candidate for real-world solar water-splitting applications. However, real-world deployment issues, such as toxic chlorine gas production and electrocatalyst poisoning effects by chlorine and other dirt particles, remains to be addressed, which is beyond the scope of the current work.

### Enhanced stability by the decoupling scheme

To explore the device stability, we evaluated the photoanode in a three-electrode PEC setup by continuous chronoamperometry under one sun illumination in neutral (1 M Na_2_SO_4_) electrolyte at 0 V vs. RHE. The initial $J_{\mathrm{H}_2}$ of the device was well retained over 150 h of operation without any significant degradation in the activity (Fig. 3e). Moreover, two-electrode PEC stability of the device is measured by short-circuiting the device vs. a Pt counter electrode and the resultant *J*_H_ was ~9.8 mA cm^−2^ and stable for <10 h, after which the current density started to decrease over time (Inset of Fig. 3e). However, adding fresh electrolyte to the system, the $J_{\mathrm{H}_2}$ recovered to the previous high of ~9.8 mA cm^−2^, revealing the stable STH over the time. The reason behind such fall and retain of $J_{\mathrm{H}_2}$ with the addition of fresh electrolyte could be due to the charge build-up in which further investigations are warranted.

X-ray photoelectron spectroscopy (XPS) analysis before and after the stability measurements (Supplementary Fig. 4a–d) shows that the surface of the metallic Ni is transformed into oxide and hydroxide forms, such as NiOx and Ni(OH)_2_, the stable forms of Ni-based OER electrocatalysts^37^. More detailed discussion on the XPS analysis is provided in the Supplementary Information.

In general, it is well known that III-V photoelectrodes undergo severe corrosion during the water-splitting process even in neutral electrolyte due to their chemical instability^17,19,21^. Despite the demonstration of high STH efficiencies, the critical issue that halts the employment of III-V materials in practical PEC systems is their poor stability^15,17,19^. Others have addressed this issue with limited success by depositing protective metal oxide layers, such as TiO_2_ using atomic layer deposition (ALD)^19^. However, the deposition of protective metal-oxides has stringent requirements and trade-offs, especially at increased thickness as it leads to parasitic light absorption and poor conductivity. In this context, it is mandatory to achieve concurrent improvement in the surface protection and electrocatalysis without parasitic light absorption that negates the efficiency. Supplementary Fig. 5 shows a literature comparison of III-V-based tandem (both double junction and triple junction) photoelectrodes (both photoanode and photocathode configurations), in which the measured pH is represented in the X-axis and the STH efficiencies are given in the Y-axis while the size of each datapoint represents the stability of the devices under the given PEC conditions^16–20,41–45^. The PEC stability demonstrated in our work is the best stability reported for any III-V-based PEC cell without compromising the efficiency, suggesting the synergistically enhanced optical, electrical, surface protection, and electrocatalysis properties made possible through our decoupling design.

In addition to the stability measurements, we evaluated the gases evolved during the continuous chronoamperometry test using an airtight syringe and analyzing the contents using gas chromatography (GC). Figure 3f shows the amount of H_2_ and O_2_ evolved from the reactor and the amount theoretically obtained by the charge passed through the electrolyte (see Supplementary Information for more details about calculations and gas measurements). After 80 min of continuous operation and collection time, we found that the average volumes of the gases were ~0.8 μL/s for H_2_ and ~0.38 μL/s for O_2_, nearly matching the theoretically calculated H_2_ and O_2_ values for a Faradaic efficiency of over 95%. The H_2_ and O_2_ gas production rates were stable over almost the entire period, indicating the device’s excellent stability without corrosion over time. We also measured the gas evolution at periodic intervals over the entire stability period, confirming the Faradic efficiencies and stability.

### Standalone wireless water-splitting

**Fig. 4:**
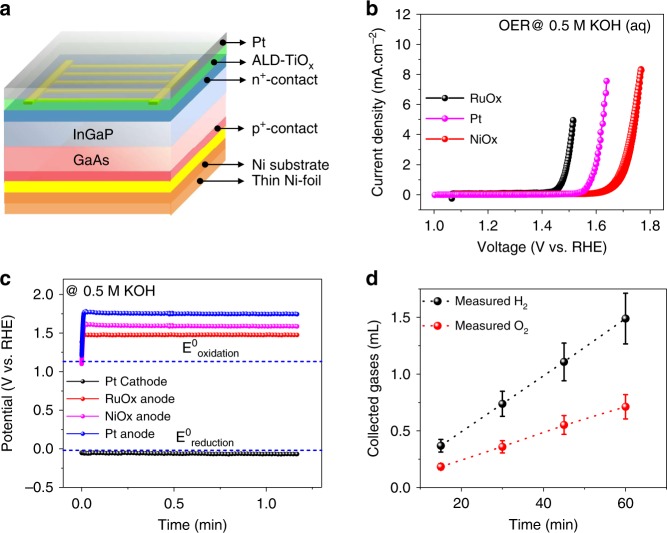
Wireless device structure and demonstration. **a** The device structure employed for the wireless configuration. **b** CV curves used to obtain the electrocatalytic overpotential values of the HER/OER catalysts for the Pt/Pt, Pt/RuOx, and Pt/NiOx electrodes in 0.5 M KOH (aq). **c** Chronopotentiometry experiments at a current density of 10 mA cm^−2^. **d** The O_2_ and H_2_ volumes measured for the device without any electrical connections

There have been multiple demonstrations of III-V-based unassisted water-splitting with efficiencies ranging from 10–19%^14,17,19,20^. In the earlier sections, we demonstrated unassisted water-splitting with an efficiency of ~9% with excellent stability. However, an unassisted-wireless device (i.e., artificial leaf), with no wires whatsoever, has yet to be achieved in III-V-based materials, mainly due to the issues in electrical conductivity of the front and back surfaces, optimum catalyst loading, surface protection, and efficient transport of protons from the anode to the cathode side (Supplementary Fig. 2). Accessing the bifacial functionality of the PEC device is important in the case of this monolithically integrated wireless design, in which both sides of the devices are in contact with the electrolyte. In our design (Fig. 4a), the successful transfer of the III-V tandem cell on the catalyst carrier substrate enables access to both the light harvesting side (which can act as a photocathode in contact with the electrolyte) and the catalyst carrier side (as a dark anode in contact with the electrolyte). Due to the highly corrosive nature of the light-absorbing III-V compounds, we opted for ALD-deposited TiO_2_ (150 nm) as a surface protection layer and Pt (5 nm) as an electrocatalyst on top of the light harvesting side. To confirm the wireless device function, we analyzed the electrocatalytic overpotentials for HER/OER with the Pt cathode using Pt, RuO_x_, and NiO_*x*_ anodes at 0.5 M KOH (aq). From the results (Fig. 4b, c), it is evident that the total potential required to drive the reaction is 1.80, 1.71, and 1.52 V vs. RHE for Pt/Pt, Pt/NiO_*x*_, and Pt/RuO_*x*_ as the HER/OER electrocatalysts, respectively. The RuO_*x*_ as the OER catalyst is unstable in alkaline conditions compared to the NiO_*x*_, although the total potential required for Pt/RuO_*x*_ as the HER/OER electrocatalyst is low. Hence, the Pt/NiO_*x*_ HER/OER catalyst combination is employed herein, which is capable of driving unassisted water-splitting with a potential of 1.9 V, which matches well with the *V*_OC_ of 2.1 V of the tandem PEC cell. Supplementary Fig. 1 provides the fabrication details of the unassisted-wireless structural design, including all the layers.

We further analyzed the device structure under 0.5 M KOH (aq) electrolyte in a sealed quartz reactor in a gas-tight environment. Once the light was turned ON, instantaneous gas bubble formation was visible, as shown in the Supplementary Information video. The evolved gases were collected and analyzed, as shown in Fig. 4d. After 30 min operation and collection, we calculated the average volumes of the gases to be ~0.41 μl/s for H_2_ and ~0.19 μl/s for O_2_, which corresponds to an STH efficiency of ~6.0%, which is thus far the highest reported value for a unassisted-wireless device i.e., “artificial leaf” configuration^46^. However, the efficiency of the unassisted-wireless device decreased by half compared to the unassisted-wired (two-electrode measurements), possibly due to poor charge carrier transport in the electrolyte and the poor conductivity of the surface protection layer (TiO_2_), which has been previously reported to reduce the efficiency in various electrolytes^21^.

In the case of unassisted-wireless operation, it is not possible to measure the current density or other similar characteristics, but only the gases evolved from the reaction. Hence, we plotted the molar ratio of the gases evolved (H_2_:O_2_) from the wireless device structure over time (Supplementary Fig. 6). The results reveal that an equimolar amount of O_2_ and H_2_ evolves during the first ~2 h of the reaction, after which the amount of O_2_ produced significantly declined, which could be attributed to the corrosion of the photoelectrode itself rather than the water-splitting reaction. It should be noted that equimolar O_2_ and H_2_ production during the initial ~2 h of the device operation is critical proof of true water-splitting, and the deviation in the latter part of the device operation (after 2 h) suggests the etching or corrosion of the photoelectrode itself instead of splitting water. We attribute this reduced stability to the exposure of the light harvesting front GaAs surface to the corrosive 0.5 M KOH (aq). Though ALD-TiO_2_ is employed here for surface protection, the PEC cell size is ~0.8 cm^2^, an area that will feature a large number of pinholes^21^, and hence the poor stability as compared to the two-electrode PEC measurements. In two- and three-electrode measurements, the front light-absorbing surface is protected by a thin-quartz film and the back-reaction surface is protected by the stable Ni-carrier substrate where OER occurs, and hence extended stability is achieved. Optimizing the ALD-TiO_2_ layer is expected to enhance the efficiency and stability of the device, though this is beyond the scope of this work.

### Mechanically flexible PEC water-splitting

An additional benefit brought about by our decoupling scheme is the device’s flexibility. The tandem PEC cell on the metal substrate is flexible and thus can be integrated onto flexible electronic modules. Flexible PEC cells must be mechanically stable, which we verified by mechanically bending the PEC cells inwards with radii of curvature from 2 to 8 mm. The results show that there was almost no change in $J_{\mathrm{H}_2}$ (varying less than 10% overall) at various bending angles (Supplementary Fig. 7a). However, there was an enormous drop in the $|V_{\mathrm{OS}} - E^o|$ of the PEC devices at radii of curvature over 2 mm. Supplementary Fig. 7b demonstrates the $|V_{\mathrm{OS}} - E^o|$ and $J_{\mathrm{H}_2}$ characteristics of the tandem PEC cell after a number of bending tests at a radius of 8 mm. The $J_{\mathrm{H}_2}$ of the PEC cell is constant after 1000 cycles of bending, demonstrating its robustness. However, the $|V_{\mathrm{OS}} - E^o|$ of the PEC cell falls from 2.10 to 1.78 V after two cycles of bending, then remaining almost constant for up to 1000 bending cycles. The fall in $|V_{\mathrm{OS}} - E^o|$, especially at higher bending angles and cycles, can be attributed to the higher strain within the functional layers, and the partially damaged catalyst carrier and finger contacts. The results demonstrate that even after high bending angles and many cycles, the flexible PEC device exhibits a $|V_{\mathrm{OS}} - E^o|$ of 1.78 V with high current density, allowing it to still be employed for unassisted PEC water splitting, although the operating potential is reduced. The resulting flexible and lightweight InGaP/GaAs tandem photoelectrode demonstrated herein is the first flexible unassisted water-splitting PEC device, featuring unprecedented stability and taking the technology a step closer to the practical enactment of III-V semiconductors for PEC water-splitting applications.

## Discussion

In summary, we have demonstrated a universal wafer-scale scheme for decoupling the light harvesting component of the photoabsorber from the surface protection and electrocatalyst components in a III-V-based PEC system to synergistically improve the device efficiency, stability, and cost. To achieve this, we employed the ELO and transfer method to produce inverted tandem InGaP/GaAs PEC cells that exhibit an STH efficiency of ~9% in a wireless configuration with an unprecedented stability of 150 h with no signs of degradation in the PEC performance, made possible by spatially decoupling the optical absorption and electrocatalysis functions. Accessing both the front and back side of the PEC device enabled by the unique ELO and transfer method allowed us to demonstrate the first fully-integrated III-V-based unassisted-wireless, with a record STH efficiency of ~6.0%. We envision that this kind of efficient, stable, and flexible standalone wireless device can be used in practical PEC hydrogen production applications.

## Methods

### Epitaxial growth of the III-V PEC cell

Epitaxial InGaP/GaAs inverted double junction was grown on 2-inch (100) GaAs with 15° misorientation towards (111) direction by employing MOCVD^34,47,48^. Trimethylgallium (TMGa), and trimethylindium (TMIn) were used as the metal-organic precursors for group III-materials whereas arsine (AsH_3_), and phosphine (PH_3_) were used as the precursors for group V-materials. First, we sequentially deposited a 0.3-μm-thick GaAs layer (Buffer 1), followed by the etch stop InGaP (0.3 μm), sacrificial layer AlAs (0.3 μm), and another etch stop InGaP (0.3 μm). Next all the active layers, including the InGaP epilayer (top junction) and GaAs epilayer (bottom junction), were deposited. The thicknesses of all the layers are given in Supplementary Fig. 1. Heavily doped n^+^-on-p^+^ GaAs with the thickness of 30 nm was deposited to act as the tunnel junction diode that connects the InGaP top cell and GaAs bottom cell by both electrically and optically. Hydrogen with the total flow of 12 L/min was maintained as the carrier gas during the deposition. The growth was carried out at the substrate temperature of 650 °C, and the reactor pressure was maintained at 60 mbar. Under the given conditions the growth rate of AlAs, GaAs, and In_0.51_Ga_0.49_P were 2.5, 1.4 and 1.4 µm, respectively.

### ELO and transfer onto a robust substrate

After the epitaxial growth by MOCVD, the device fabrication and ELO processes were performed as described earlier^34,47,48^. The steps involved in the ELO and transfer process is shown in the Supplementary Fig. 1. In simple terms, AuBe/Au with the thickness of 50/150 nm was deposited on the fresh epitaxial layer to act as both the seed layer for electroplating of metals and the protection mask during the dry etching process. For dry etching we employed the inductively coupled plasma-reactive ion etching with a cross-pattern mask to etch the epilayer to expose the Buffer 2. Photoresist was filled immediately on the etched hole to protect the side-wall etching during the ELO process. Then, the metallic Ni with the thickness of ~80 μm was electroplated on the AuBe/Au surface, followed by the etching of photoresist with an acetone to form a through-hole. The etched through-hole length and width were ~5.12 and ~0.62 mm, respectively. Then the sample was immersed in a solution containing HF:acetone (1:1 ratio) for the removal of AlAs sacrificial layer, leading to the separation of the single-crystalline GaAs substrate and light-absorbing epilayers. Diluted HCl (30%) was used to expose the clean epilayer and remove the amorphous As-oxide films formed during the ELO process. The AuGe/Au film (50/150 nm) was deposited on the *n*^+^GaAs contact layer as the front grid electrode. Finally, NH_4_OH:H_2_O_2_:H_2_O (1:9:40) was used to remove the n^+^-GaAs contact layer. Finally, the peeled off structure was successfully transferred to the thin Ni foil (0.125 mm thickness).

### PEC device fabrication

Ohmic contact was achieved by applying the Ga-In eutectic on the top finger electrode without shorting the PEC device and a Cu thin foil contact lead was taken out. The device is covered with an epoxy (Loctite Hysol 1 C). The Cu thin foil was then threaded through a 7 mm-diameter glass tube, and the PEC cell was affixed to one end with epoxy. Overall, the front surface is protected with a thin quartz slide, whereas the bottom Ni-thin foil (0.125 mm) is exposed to the electrolyte to carry out the OER reaction. Ni-thin foil was employed as the carrier for the peeled and transferred structure due to multiple advantages as follows. Metals are preferred as the handling materials due to their facile transfer of photoinduced carriers (holes for photoanodes) from the semiconductor surface to the electrolyte. Flexible carrier choices, such as metals, films, or even plastics, allow unique pathways for the simultaneous protection of the semiconductor electrode with excellent electrocatalytic properties from corrosive electrochemical conditions. Moreover, while immersing the 0.5 M KOH during the OER reaction the surface of the Ni-thin foil is expected to form NiOOH, Ni(OH)_2_ and other oxide forms that facilitates a superior OER^26,49,50^.

### Wireless device fabrication

In case of the unassisted-wireless device, the top light-absorbing surface with finger electrodes was covered with 150 nm of ALD-TiO_2_ followed by the deposition of 5 nm of Pt, which acts as the HER electrocatalyst. The bottom side of the device was maintained similar to the configuration employed in the two- and three-electrode measurements, in which the thin Ni-foil was exposed to the 0.5 M KOH electrolyte. The reaction was carried out by fully immersing the 0.8 cm^2^ of the fabricated wireless structure into the electrolyte in a closed reactor. The device was directly illuminated with AM 1.5 G one-sun light and the gases were collected and analyzed over time using gas chromatography (Agilent-7890B GC).

### Solar-fuels modeling

The ideal bandgap combinations for achieving the maximum STH efficiency of the PEC devices have been estimated previously. Initial calculations consider only the theoretical thermodynamic splitting potential of water (1.23 V at 25 °C), and is aimed at achieving maximum current density^51,52^. More comprehensive assessments including the electrocatalytic and system overpotentials are considered by some of the later literature^27,39^. Recently detailed balance calculations of practical STH for tandem absorbers with various parameters such as optical loss due to the electrolyte, and other implications are considered^53^. Here we have used the most advanced simulations based on the recent modeling by including all the electrocatalytic and system overpotentials^54^.

### Incident photon-to-current efficiency

We measured the incident photon-to-current efficiency (IPCE) under monochromatic illumination^14^. Such analytical measurements can give meaningful insights into the contribution of surface treatments to the conversion of incident photons into charge carriers. The IPCE was determined in a short-circuit condition vs. Pt without any applied bias, which is expected to reveal the true STH of the device^14^:

2 $$\mathrm{IPCE}(\% ) = \frac{{1240 \times J_{\mathrm{ph}}(\mathrm{mA}/\mathrm{cm}^2)}}{{\lambda (\mathrm{nm}) \times P_{\mathrm{light}}(\mathrm{mW}/\mathrm{cm}^2)}} \times 100\%$$

in which *λ* is the wavelength of the illuminating monochromatic photons, and *P*_light_ is the illuminating light power density at a given wavelength.

### X-ray photoelectron spectroscopy

Additional characterization by XPS was used to identify the elemental composition and oxidation states of Ni/GaAs/InGaP before and after electrochemical cycling. Spectral fitting of Ni, oxide, and hydroxide spectral components of the XPS data measured for systems containing all three species is shown in Supplementary Fig. 4^55,56^. Supplementary Fig. 4a, b shows the high resolution Ni2p XPS spectra of the Ni carrier/electrocatalyst layer before and after the PEC stability measurements, consisting of three major components, including Ni, NiO_*x*_, and Ni(OH)_2_^48^. The results reveal that: (i) the main binding energy of the Ni 2p_3/2_ peak in the Ni/GaAs/InGaP sample occurs at 852.6 eV, which belongs to metallic Ni^0^. Additionally, the peaks at 853.8 eV (Ni(II)) and 855.6 eV (Ni(III)) can be attributed to NiO_*x*_ and Ni(OH)_2_. (ii) After the PEC testing, the main binding energy peaks of Ni 2p_3/2_ are from NiO_x_ (853.8 eV and 855.6 eV), with no traces of metallic Ni^0^ peak, revealing the successful electrochemical activation of remenant Ni in 0.5 M KOH (aq) electrolyte and the formation of Ni(OH)_2_ and NiO_*x*_ passive films^50^. Supplementary Fig. 4c, d shows the O 1s spectra before and after PEC characterization, respectively. Post PEC testing of the O 1s peak can be coherently fit by two Gaussian components with binding energies at 529.7 eV and 531.2 eV, corresponding to the oxygen states in Ni(OH)_2_ and NiO_*x*_, demonstrating that the post-cycled electrode surface is primarily composed of Ni hydroxide/oxyhydroxide^57^.

### STH calculations

The unassisted-wireless device experiments were carried out at one sun intensity under 0.5 M KOH electrolyte with no electrical connections, and hence it is not possible to measure the current density and efficiency. The reaction was performed in the quartz reactor, and the gases were collected using a gas tight syringe and analyzed immediately through the GC. All the efficiencies reported here are based on the following equation.

$$\mathrm{STH} = \left[ {\frac{{\left( {\mathrm{mmol}\frac{{\mathrm{H}_2}}{\mathrm{s}}} \right) \times \left( {237\frac{{\mathrm{KJ}}}{{\mathrm{mol}}}} \right)}}{{P_{\mathrm{total}}\left( {\frac{{\mathrm{mW}}}{{\mathrm{cm}^2}}} \right) \times \mathrm{Area}\;\left( {\mathrm{cm}^2} \right)}}} \right]_{\mathrm{AM}\;1.5G}$$

## Supplementary information

Supplementary Information

Video

## Data Availability

The data that support the plots within this paper and other finding of this study are available from the corresponding author upon reasonable request.
