# Supplementary material for: An efficient and stable photoelectrochemical system with 9% solar-to-hydrogen conversion efficiency via InGaP/GaAs double junction
Source: Nat Commun. 2019 Nov 21;10:5282. doi: 10.1038/s41467-019-12977-x (PMC6872648; doi:10.1038/s41467-019-12977-x)
Supplement: Supplementary file 1 — Supplementary Information [file 41467_2019_12977_MOESM1_ESM.pdf]

Supplementary Information

An efficient and stable photoelectrochemical system with 9% solar-to-hydrogen conversion efficiency via InGaP/GaAs double junction

Purushothaman Varadhan,[1,2,†] Hui-Chun Fu,[1,2,†] Yu-Cheng Kao,[3] Ray-Hua Horng,[4,5] and Jr-Hau He[1,2,6,*]

[1]*Computer, Electrical, and Mathematical Sciences and Engineering, King Abdullah University of Science and Technology, (KAUST), Thuwal 23955-6900, Saudi Arabia.*

[2]*KAUST Solar Center, KAUST, Thuwal 23955-6900, Saudi Arabia.*

[3]*Graduate Institute of Precision Engineering, National Chung Hsing University, Taichung 402, Taiwan, ROC.*

[4]*Institute of Electronics, National Chiao Tung University, Hsinchu 300, Taiwan, ROC.*

[5]*Center for Emergent Functional Matter Science, National Chiao Tung University, Hsinchu 300, Taiwan, ROC.*

[6]*Department of Materials Science and Engineering, City University of Hong Kong, Kowloon, Hong Kong SAR.*

[†]These authors contributed equally.

[*]Corresponding author: jrhauhe@cityu.edu.hk

Supplementary Figures

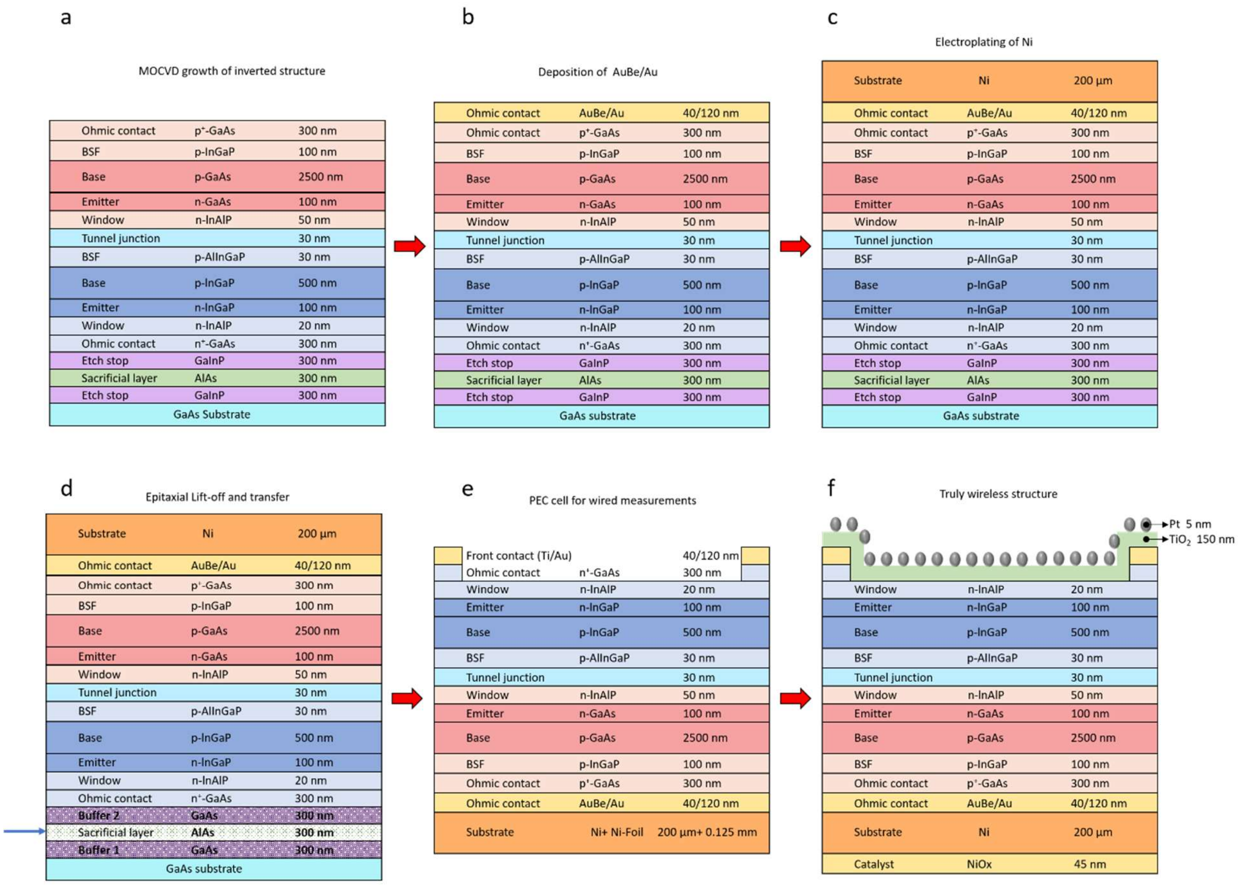

Supplementary Figure 1| The wireless device fabrication steps, including all the layers, contacts, and electrocatalysts, with their thicknesses indicated. **a,** The as-grown epitaxial layer on top of the GaAs substrate. **b-d,** The ELO and transfer steps. **e,** The device structure used for the two- and three-electrode measurements. **f,** The final, unassisted-wireless structure i.e., artificial leaf structure.

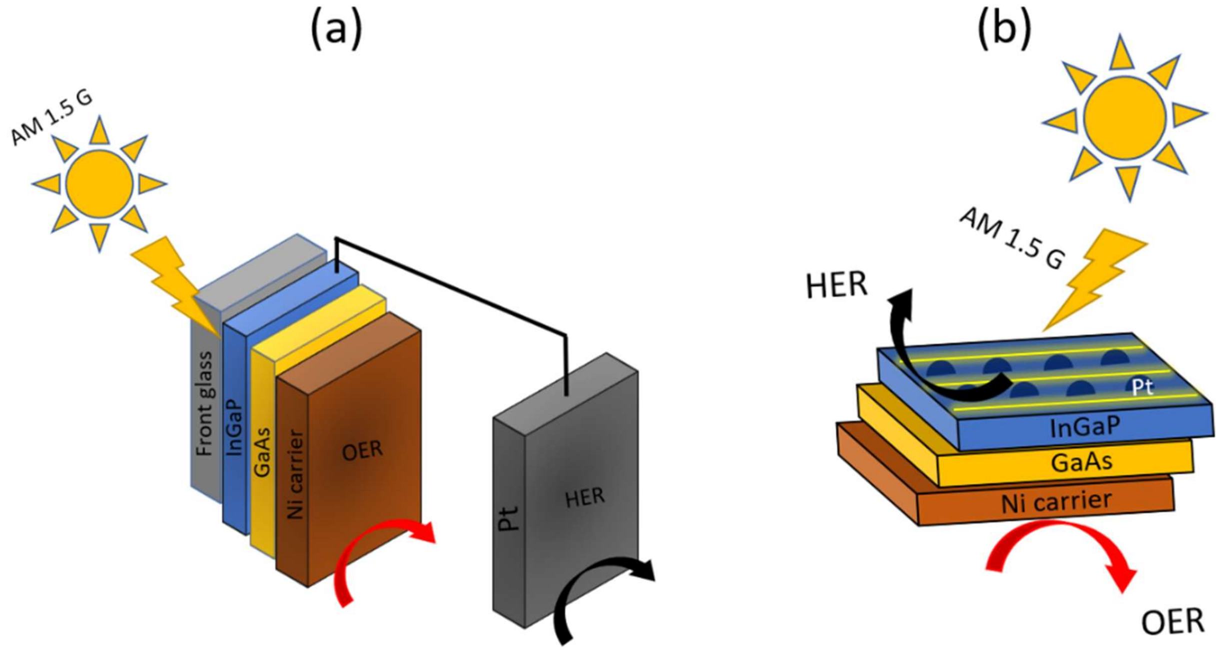

Supplementary Figure 2| (a) Unassisted-wired configuration, and (b) Unassisted-wireless configuration. In unassisted-wired configuration the working electrode (WE) is connected to the counter electrode (CE) through an external wire (controlled by the potentiostat). In this configuration without any external bias the photon exited holes carry out the oxygen evolution reaction (OER), whereas the electrons are diverted to the CE for hydrogen evolution reaction (HER). In unassisted-wireless configuration (artificial leaf configuration) the photon excited holes and electron move towards the OER and HER surface for the oxidation and reduction reactions.

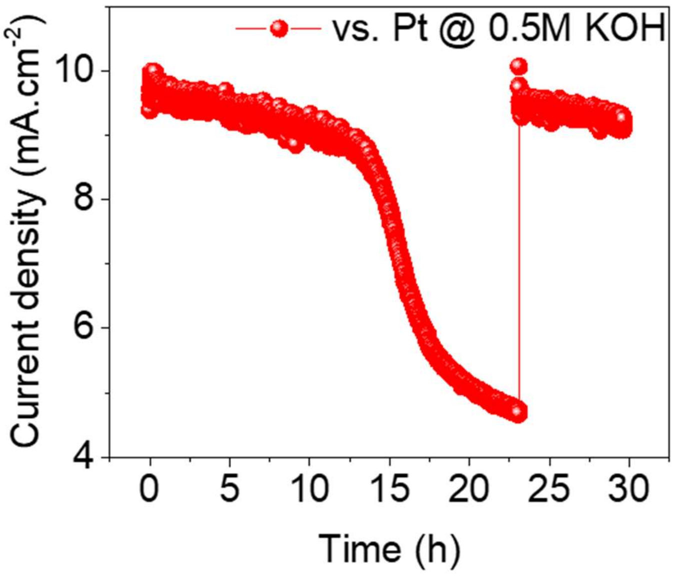

Supplementary Figure 3| The J_{H_2} vs. Pt counter electrode of the thin-Ni foil protected InGaP/GaAs double junction photoanode (unassisted-wired configuration). The J_{H_2} falls after ~10 h. However, the addition of fresh electrolyte caused the current density to recover.

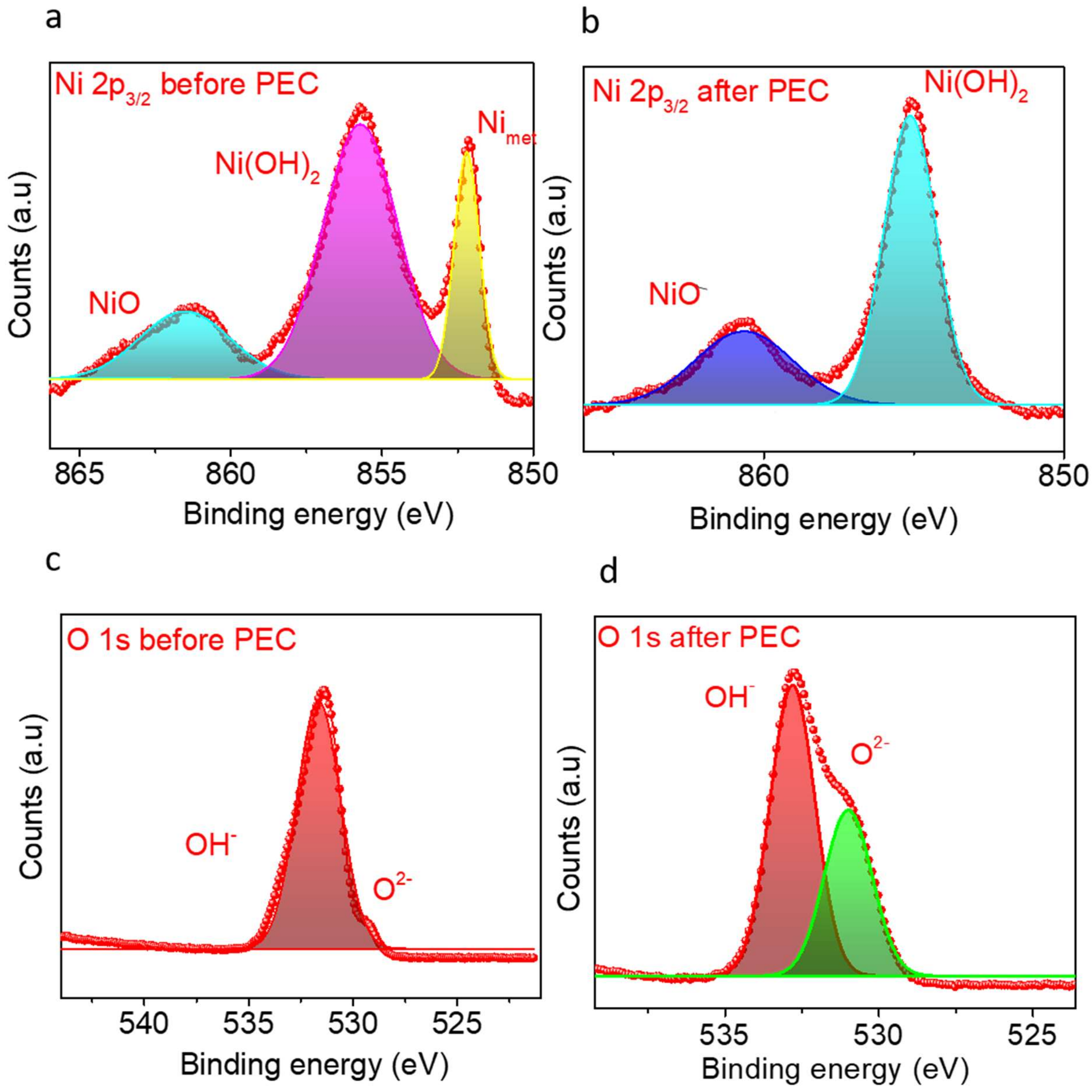

Supplementary Figure 4| X-ray photoelectron spectroscopy. **a, b,** High resolution Ni2p XPS spectra and **c,d,** high resolution O1s spectra of the Ni carrier/electrocatalyst layer before and after the 150 h photoelectrochemical stability measurements, respectively.

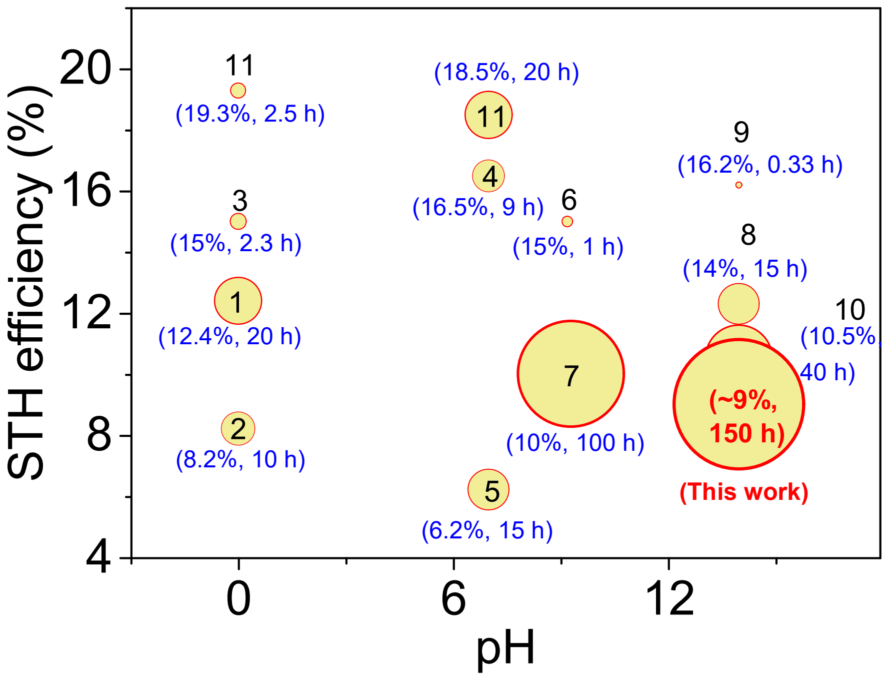

Supplementary Figure 5| Literature comparison of the measured pH (x-axis) vs. STH efficiency (Y-axis) of III-V based double and triple junction PEC water splitting cells[1-11]. The size of each datapoint represents the stability of the devices under the given PEC conditions. The numbers in parentheses represent their corresponding efficiency and stability in hours.

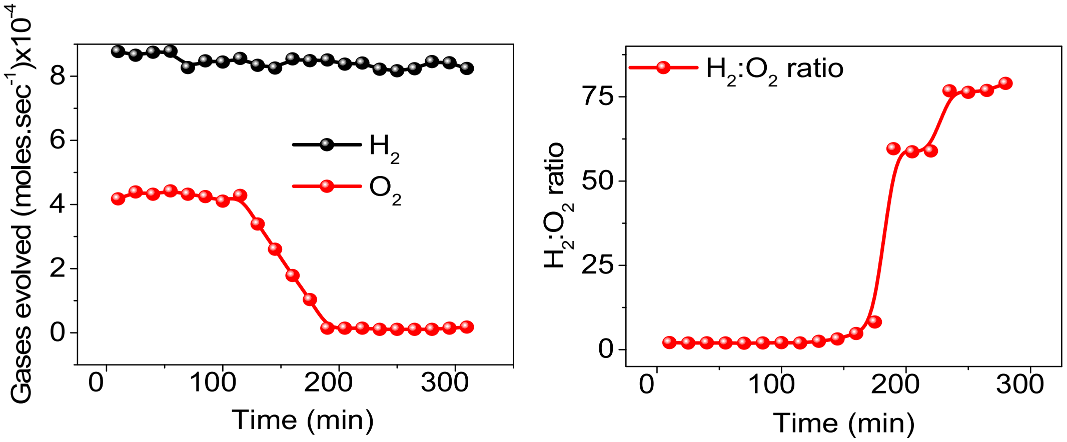

Supplementary Figure 6| a. Gases evolved from the wireless device structure over time under AM 1.5G one sun illumination. **b.** The H_2 to O_2 ratio over time, calculated from the gases evolved from the PEC reaction. It should be noted that the hydrogen to oxygen ratio is ~2 during the initial period of time (~ 3 hours) after which the oxygen evolution almost goes to zero which can be attributed to the oxidation/corrosion of the semiconductor itself rather than the water oxidation reaction.

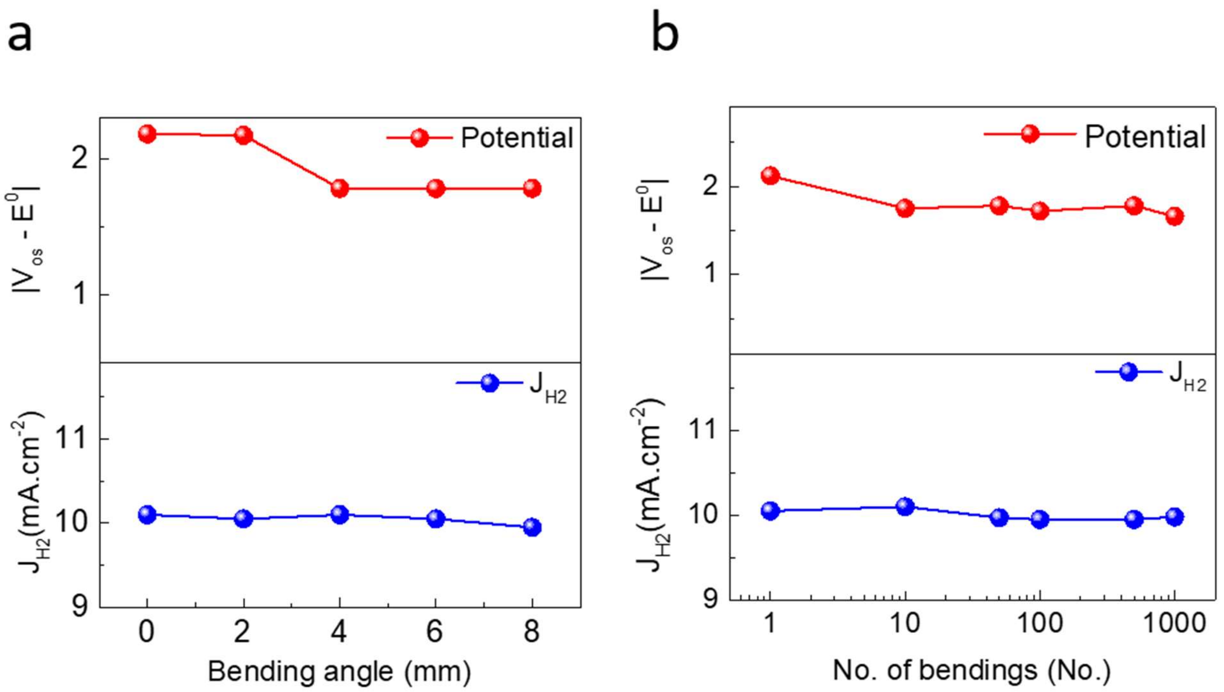

Supplementary Figure 7| Mechanically flexible PEC water splitting, a, PEC performance characteristics of the flexible InGaP/GaAs photoanode at different bending angles. **b,** The corresponding device performance at different bending cycles measured under a three-electrode setup in 0.5 M KOH(aq) under one sun illumination. The results demonstrate the sturdiness of the sample even with high bending angles and bending cycles (8 cm and 1000 cycles, respectively).

Supplementary Table 1| Comparison of unassisted wireless PEC devices demonstrated thus far in literature

Device structure	Electrolyte used	STH (%)	Stability (h)	Supplementary References
Tandem 2J-InGaP/GaAs	0.5M KOH	6.0	4	This work
Tandem 2J-InGaP/GaAs	0.5M KOH	9.0	4	10
Triple junction-a-Si	1M H_2SO_4	5.0	6	12
Triple junction -a-Si	1M KBO4	2.5	0.2	13
Triple junction -a-Si	5M H2SO4	2.6	-	14
Double junction a-Si + WO_3	1M H2SO4	0.7	10	15
Series connected CIS	1M KOH	3.1	-	16
$CH_3NH_3PbI_3$ perovskite single junction solar cell with $BiVO_4$	0.1M KCi	3.0	12	17

Supplementary References

1. Khaselev, O. & Turner, J. A. A monolithic photovoltaic-photoelectrochemical device for hydrogen production via water splitting. *Science* **280**, 425-427 (1998).
2. Kainthla, R. C., Zelenay, B. & Bockris, J. O. Significant efficiency increase in self-driven photoelectrochemical cell for water photoelectrolysis. *J. Electrochem. Soc.* **134**, 841-845 (1987).
3. Peharz, G., Dimroth, F. & Wittstadt, U. Solar hydrogen production by water splitting with a conversion efficiency of 18%. *Int. J. Hydrogen Energ.* **32**, 3248-3252 (2007).
4. Khaselev, O., Bansal, A. & Turner, J. A. High-efficiency integrated multijunction photovoltaic/electrolysis systems for hydrogen production. *Int. J. Hydrogen Energ.* **26**, 127-132 (2001).
5. Modestino, M. A. *et al.* Robust production of purified H_2 in a stable, self-regulating, and continuously operating solar fuel generator. *Energ. Environ. Sci.* **7**, 297-301 (2014).
6. Fujii, K. *et al.* Characteristics of hydrogen generation from water splitting by polymer electrolyte electrochemical cell directly connected with concentrated photovoltaic cell. *Int. J. Hydrogen Energ.* **38**, 14424-14432 (2013).
7. Sun, K. *et al.* A stabilized, intrinsically safe, 10% efficient, solar-driven water-splitting cell incorporating earth-abundant electrocatalysts with steady-state pH gradients and product separation enabled by a bipolar membrane. *Adv. Energy Mater.* **6**, 1600379 (2016).
8. May, M. M., Lewerenz, H. J., Lackner, D., Dimroth, F. & Hannappel, T. Efficient direct solar-to-hydrogen conversion by in situ interface transformation of a tandem structure. *Nat. Commun.* 6, 8266 (2015).
9. Young, J. L. *et al.* Direct solar-to-hydrogen conversion via inverted metamorphic multi-junction semiconductor architectures. *Nat. Energy* 2, 17028 (2017).
10. Verlage, E. *et al.* A monolithically integrated, intrinsically safe, 10% efficient, solar-driven water-splitting system based on active, stable earth-abundant electrocatalysts in conjunction with tandem III-V light absorbers protected by amorphous TiO_2 films. *Energ. Environ. Sci.* **8**, 3166-3172 (2015).
11. Cheng, W. H. *et al.* Monolithic photoelectrochemical device for direct water splitting with 19% efficiency. *ACS Energy Lett.* 3, 1795-1800 (2018).
12. Lin, G. H., Kapur, M., Kainthla, R. C. & Bockris, J. O. M. One step method to produce hydrogen by a triple stack amorphous silicon solar cell. *Appl. Phys. Lett.* **55**, 386–387 (1989).
13. Reece Steven, Y. et al. Wireless solar water splitting using silicon-based semiconductors and earth-abundant catalysts. *Science* **334**, 645-648 (2011).
14. Appleby, A. J. *et al.* An amorphous silicon based one unit photovoltaic electrolyzer. *Energy* **10**, 871-876 (1985).
15. Miller, E., Paluselli, D. Marsen, B. & Rocheleau R. E. Development of reactively sputtered metal oxide films for hydrogen-producing hybrid multijunction photoelectrodes, *Sol. Energy Mater Sol. Cells* **88**, 131-144 (2005).
16. Kim, S. *et al*. A highly versatile and adaptable artificial leaf with floatability and planar compact design applicable in various natural environments. *Adv. Mater.* **29**, 1702431 (2017).
17. Kim, J. H. *et al*. Wireless solar water splitting device with robust cobalt-catalyzed, dual-doped $BiVO_4$ photoanode and perovskite solar cell in tandem: a dual absorber artificial leaf. *ACS Nano* **9**, 11820-11829 (2015).
